# Ion–Molecule Reaction Products as Probes and Precursors for Preparative Mass Spectrometry

**DOI:** 10.1002/chem.202503569

**Published:** 2026-01-29

**Authors:** Markus Rohdenburg, Sebastian Kawa, Kay‐Antonio Behrend, Harald Knorke, Jonas Warneke

**Affiliations:** ^1^ Wilhelm‐Ostwald‐Institut Für Physikalische und Theoretische Chemie Universität Leipzig Leipzig Germany; ^2^ Leibniz Institute of Surface Engineering (IOM) Leipzig Germany

**Keywords:** gas‐phase reactions, ion soft‐landing, ion–molecule reactions, mass spectrometry, surface chemistry

## Abstract

The investigation of ion–molecule reactions in mass spectrometers (i.e., bond formation between a reactive gaseous ion and a neutral reagent) is a well‐established method for analyzing fundamental ion reactivity. However, the potential of such reactions to generate new ions used in preparative mass spectrometry has only recently come into focus. This concept article highlights the dual significance of ion–molecule reaction products in preparative mass spectrometry: First, they provide valuable insights into the intrinsic reactivity and selectivity of reactive ions in the gas phase, forming the foundation of their reactions at molecular interfaces during ion deposition. Second, the gaseous products themselves can be deposited as mass‐selected building blocks for synthesizing thin films and functional materials. Herein, we discuss opportunities and challenges involved in translating knowledge from the gas‐phase chemistry of reactive ions to interface reactivity. This includes considerations such as the comparability of reaction partners, changes in the nature of ions upon deposition, the emergence of new reaction pathways in condensed phases, and orientation effects at interfaces. A mechanistic understanding of the gas‐phase reactions of reactive ions, as well as the mass‐selected deposition of their products, is opening up new possibilities in surface functionalization and thin‐layer synthesis.

## Graphical abstract

Ion–molecule reactions in the gas phase of mass spectrometers are important for preparative mass spectrometry because they (1) provide knowledge about the intrinsic reactivity of ions, helping to predict and control their reactivity at interfaces, and (2) generate new complex ions that can be mass selected and deposited on surfaces.

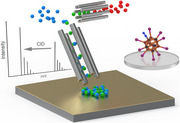

## 1 Introduction

Ion–molecule reactions in mass spectrometers are an important tool of analytical and physical chemistry, which are used to probe bond formation with ions on a fundamental level, independent of condensed phase effects such as solvation or counterion coordination [1, 2]. Coupled with gas‐phase spectroscopic methods and computational chemistry, structures and dynamics in complex molecular systems can be elucidated, for example, elementary reactions at active sites of model catalysts [3, 4]. The isolation of reaction partners in a vacuum, the control of ions in electric fields, and the mass spectrometric detection of reactants and products dependent on various parameters allow for a high degree of reaction control and enable detailed mechanistic insights. In addition to elucidating fundamental reaction mechanisms, further applications can be found in astrochemistry to investigate the formation of the first molecular building blocks of life [5], or in environmental sciences to understand atmospheric processes in the ionosphere [6].

**SCHEME 1:**
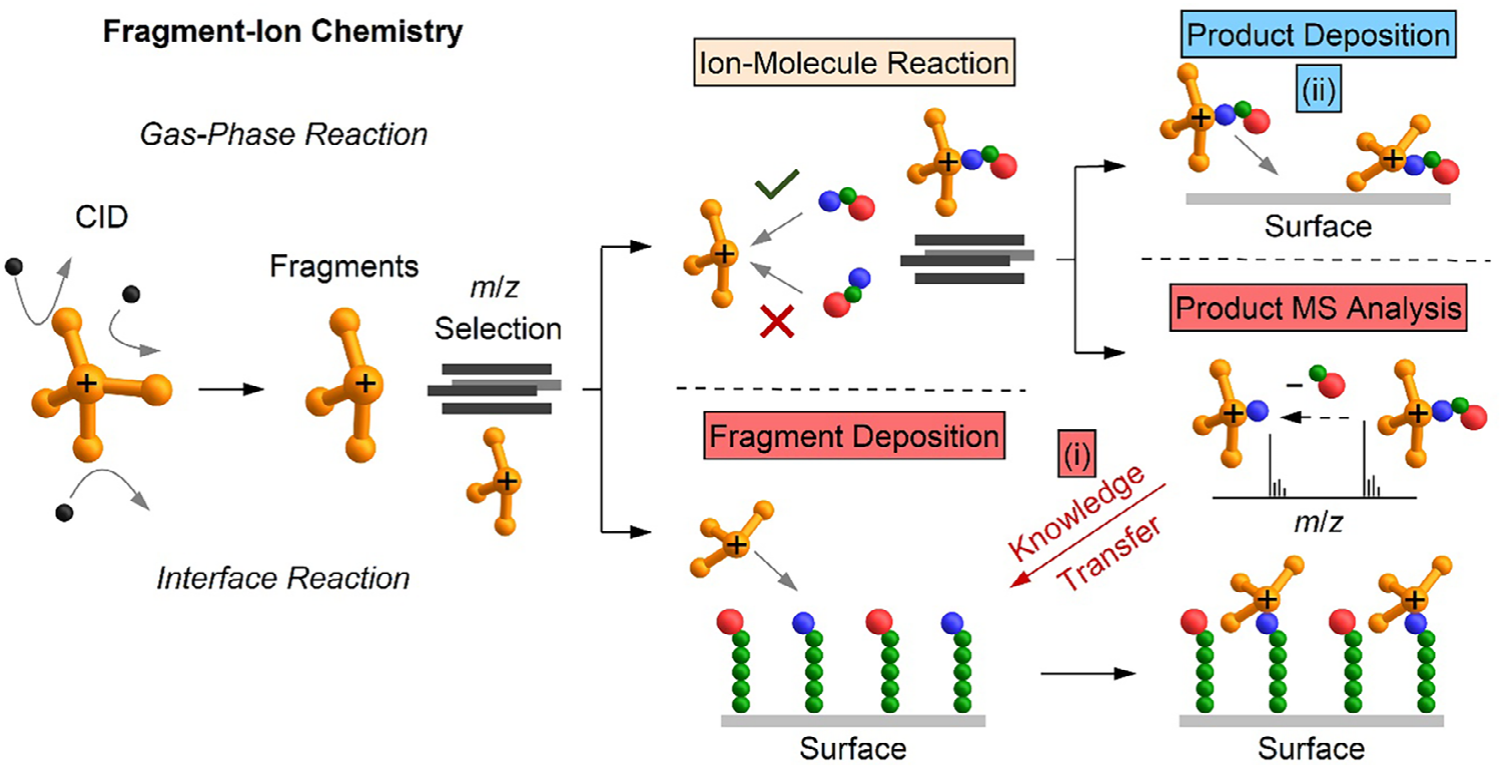
Two different roles that ion–molecule reactions play in preparative mass spectrometry: (i) Understanding reactions and binding modes of reactive ions in the gas phase in order to transfer knowledge of fundamental reactivity and to predict reactions at molecular interfaces, and (ii) direct deposition of ion–molecule reaction products for the generation of surface layers of novel ionic products.

The importance of ion–molecule reactions for such investigations is unquestioned, but the products observed in mass spectra are usually not considered as potential building blocks for new bulk material. However, preparative mass spectrometry (MS) enables the transfer of gaseous ions into the condensed phase [7, 8, 9, 10, 11, 12]. Electrospray (ESI)‐based preparative MS using *m*/*z* filtering enables deposition of selected complex ions [12, 13]. Prior to 2016, most studies focused on fundamental investigations of isolated ions on surfaces or in cryogenic matrices [12, 13, 14]. In recent years, ESI‐based deposition has rapidly developed in two different research branches: (1) The deposition of individual biomolecules on cryogenically cooled substrates (in some cases under preservation of their (almost) native structure) allows their imaging with a near‐atomic resolution using advanced microscopy. This opens new perspectives in structural biology [15, 16, 17]. (2) High ion current soft‐landing instruments enable the deposition of macroscopic layers using ions from the gas phase [18, 19, 20, 21, 22]. Due to the implementation of collision cells into such instruments [18], reactive fragment ions become available as reagents for thin‐layer synthesis on surfaces. The field of molecular layer synthesis with mass‐selected fragment ions has recently been reviewed and opens new possibilities to study reactive intermediates at interfaces and for the preparation of thin functional films [23]. This concept article focuses on the products of ion–molecule reactions of reactive fragment ions in the gas phase and their importance for layer synthesis on surfaces. We address two aspects (see Scheme 1):

- *Mechanistic insights from gas‐phase reactions*: Reactions of fragment ions in the gas phase of mass spectrometers are usually expected to provide information about the intrinsic reactivity of the probed ions. We discuss the transferability (and its limitations) of gas‐phase reactivity to the reactivity of the ions in deposition experiments.
- *Direct deposition of products*: The products of ion–molecule reactions can be mass‐selected and deposited on surfaces. This approach was already previously proposed to advance preparative mass spectrometry [24], but the first experimental results were published only very recently.

## 2 Ion–Molecule Reactions in the Gas Phase as a Probe for Reactions at Interfaces

If the fragmentation of a covalent bond in a stable precursor ion yields an undercoordinated atom and the vacant site is not filled up via an intramolecular rearrangement, the resulting fragment ion can be considered as reactive. Some general, rather intuitive rules of thumb have been proposed to evaluate the reactivity of a fragment ion for deposition experiments: (A) **Precursor stability determines fragment reactivity**: The more thermodynamically stable the precursor ion is and the more energy is required to cleave a ligand/substituent in the gas phase (larger dissociation enthalpy), the more reactive is the resulting vacant site of the fragment ion, both in the gas phase and upon deposition on surfaces. (B) **Reactivity similarities in the gas phase and at interfaces**: If the fragment ion reacts with a functional group of a reagent in an ion–molecule reaction in the gas phase, then also bond formation with similar functional groups can be expected on the surface.

Although these intuitive rules were confirmed for several example cases [25, 26, 27], they can be misleading if certain restrictions are not taken into account. In order to understand the relationship between the reactivity of fragment ions in the gas phase and on a surface, we will discuss the reaction behavior of a range of fragment ions, from moderately reactive to super‐reactive, in relation to the assumptions (A) and (B).

**FIGURE 1:**
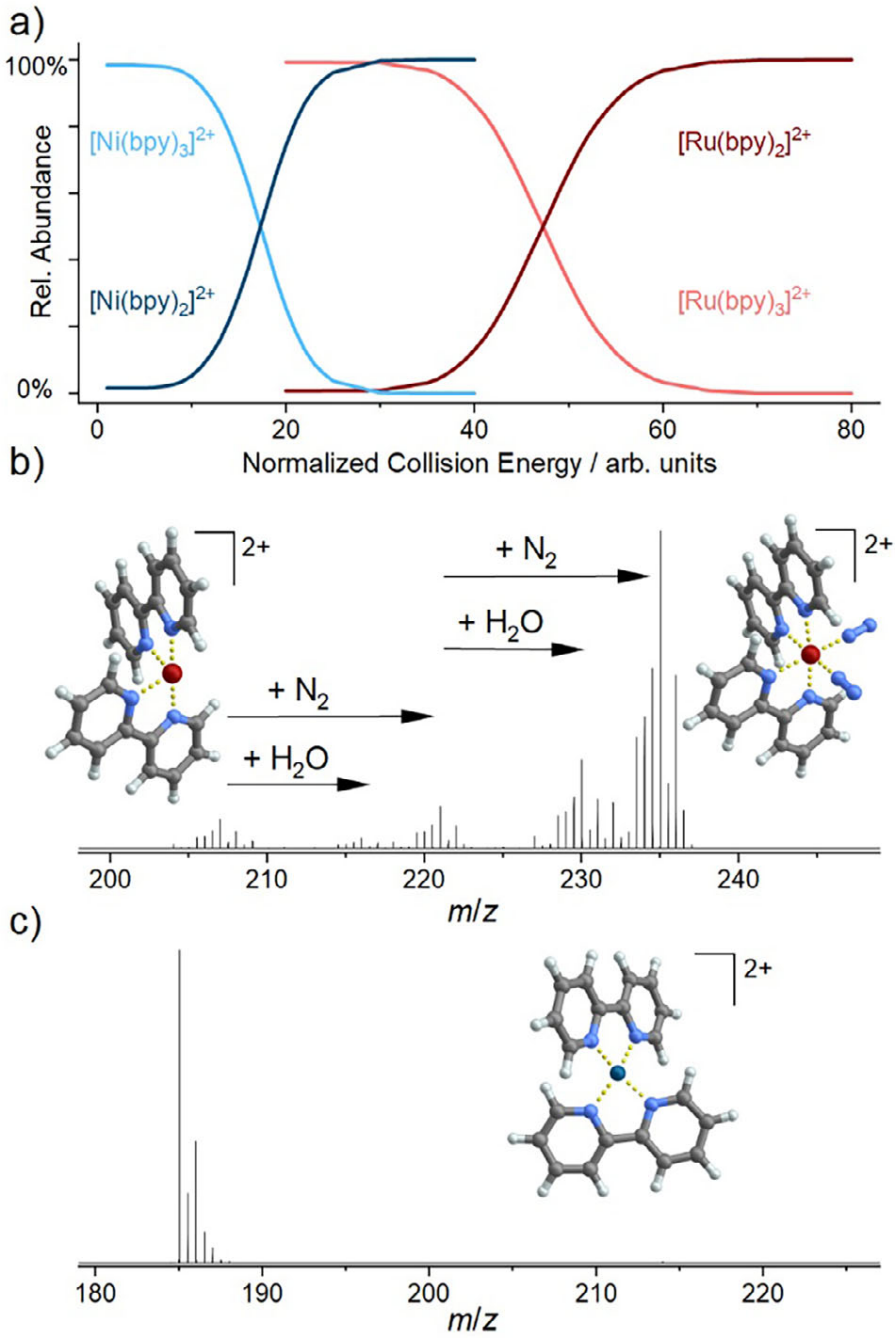
Results of higher‐energy collision‐induced dissociation (HCD) of the two organometallic dications [Ru(bpy)_3_]^2+^ and [Ni(bpy)_3_]^2+^ (bpy = bipyridine): (a) Fragmentation efficiency curves showing relative abundances of precursor and fragment ions (after elimination of one bpy ligand) as a function of normalized collision energy. For [Ru(bpy)_2_]^2+^, the intensity of the gas phase adducts and the bare fragment was summed up. (b) Mass spectrum acquired after isolation and fragmentation of [Ru(bpy)_3_]^2+^ (CE = 50 arb. units) showing the [Ru(bpy)_2_]^2+^ (*m*/*z* 207) fragment ion and the products of ion–molecule reactions with H_2_O and N_2_ present in the background gas. (c) Mass spectrum acquired after isolation and fragmentation of [Ni(bpy)_3_]^2+^ (CE = 17 arb. units) showing the [Ni(bpy)_2_]^2+^ (*m*/*z* 185) fragment ion. No ion–molecule reaction products were observed.

Collision‐induced dissociation (CID) of the ions [Ni(bpy)_3_]^2+^ and [Ru(bpy)_3_]^2+^ (bpy = bipyridine) results in elimination of a bpy ligand. Fragmentation efficiency curves are shown in Figure 1a and compare the intensities of the precursor ion and the fragment ion formed by CID, dependent on the normalized collision energy (CE, vendor‐specified arbitrary unit) [28]. In agreement with computational results [29, 30], bpy elimination from the Ni precursor ion occurs at much lower excitation energy than from the Ru precursor ion. The fragment ions [Ru(bpy)_2_]^2+^ and [Ni(bpy)_2_]^2+^ exhibit very different reaction behavior toward the residual gases of the mass spectrometer, as compared in Figure 1b, c: [Ru(bpy)_2_]^2+^ binds up to two molecules of water and/or nitrogen, but no adduct formation is detected in the case of [Ni(bpy)_2_]^2+^. The different reactivity of the two fragment ions can be explained by their energetically preferred geometry. [Ni(bpy)_2_]^2+^ relaxes into a near square‐planar structure upon ligand loss, while the Ru complex remains in a quasi‐octahedral geometry with two vacant binding sites. The differences in precursor stability are also reflected in ion deposition experiments: [Ru(bpy)_3_]^2+^ is structurally stable when soft‐landed on surfaces. In contrast, dissociation products of [Ni(bpy)_3_]^2+^ (i.e., [Ni(bpy)_2_]^2+^ and [Ni(bpy)]^2+^) are detected in high abundance following deposition. Ex situ MS analysis of layers formed by deposition of [Ru(bpy)_2_]^2+^ revealed a variety of reaction products with trace contaminants (e.g., inorganic anions, carboxylates, and sulfonates with various organic residues) that may originate from the surface and/or the solvent used for MS analysis [31]. In contrast, layers containing [Ni(bpy)_2_]^2+^ did not show such corresponding adducts. However, if deuterium‐labeled bpy was added to the solvent used for MS analysis of the layer, binding of this ligand to the Ni center was observed, demonstrating that [Ni(bpy)_2_]^2+^ is still reactive toward strongly binding reagents [32]. The described reactivity differences of Ru and Ni bpy complexes apparently confirm the correlation between the energy required for fragment formation and the reactivity of the fragments in the gas phase and on surfaces, although it is obvious that aspects like geometry rearrangements must also be taken into account.

Reactions with H_2_O and N_2_ (typically used as a collision gas or present in the background) are frequently observed for reactive cationic fragments within mass spectrometers. Comparatively higher reaction rates for binding these molecules are often correlated with a higher reactivity of the probed cation in general, that is, also toward reaction partners on the surface. If even unreactive nitrogen is bound by a fragment ion, it can usually be expected that this ion reacts immediately on surfaces with available reagents and is difficult to stabilize, while ions showing no adduct formation in the gas phase may exhibit longer lifetimes on the surface. However, there is **restriction 1 (comparability of reaction partners)**: A correlation between the required energy for fragmentation and the reactivity of the fragment ion can only be expected if the reaction partner is “related” in its chemical nature to the cleaved ligand. bpy would act as a Lewis base/nucleophile. H_2_O and N_2_ in the gas phase, as well as carboxylates, sulfates, and inorganic anions in the condensed phase, react as bases as well, therefore fulfilling the mentioned condition, and are thus able to perform a “reverse reaction” with respect to the fragmentation reaction. In contrast, the reactivity of a fragment ion toward electrophiles or radicals on the surfaces cannot be predicted only from reactions with H_2_O and N_2_ within the gas phase of mass spectrometers.

**FIGURE 2:**
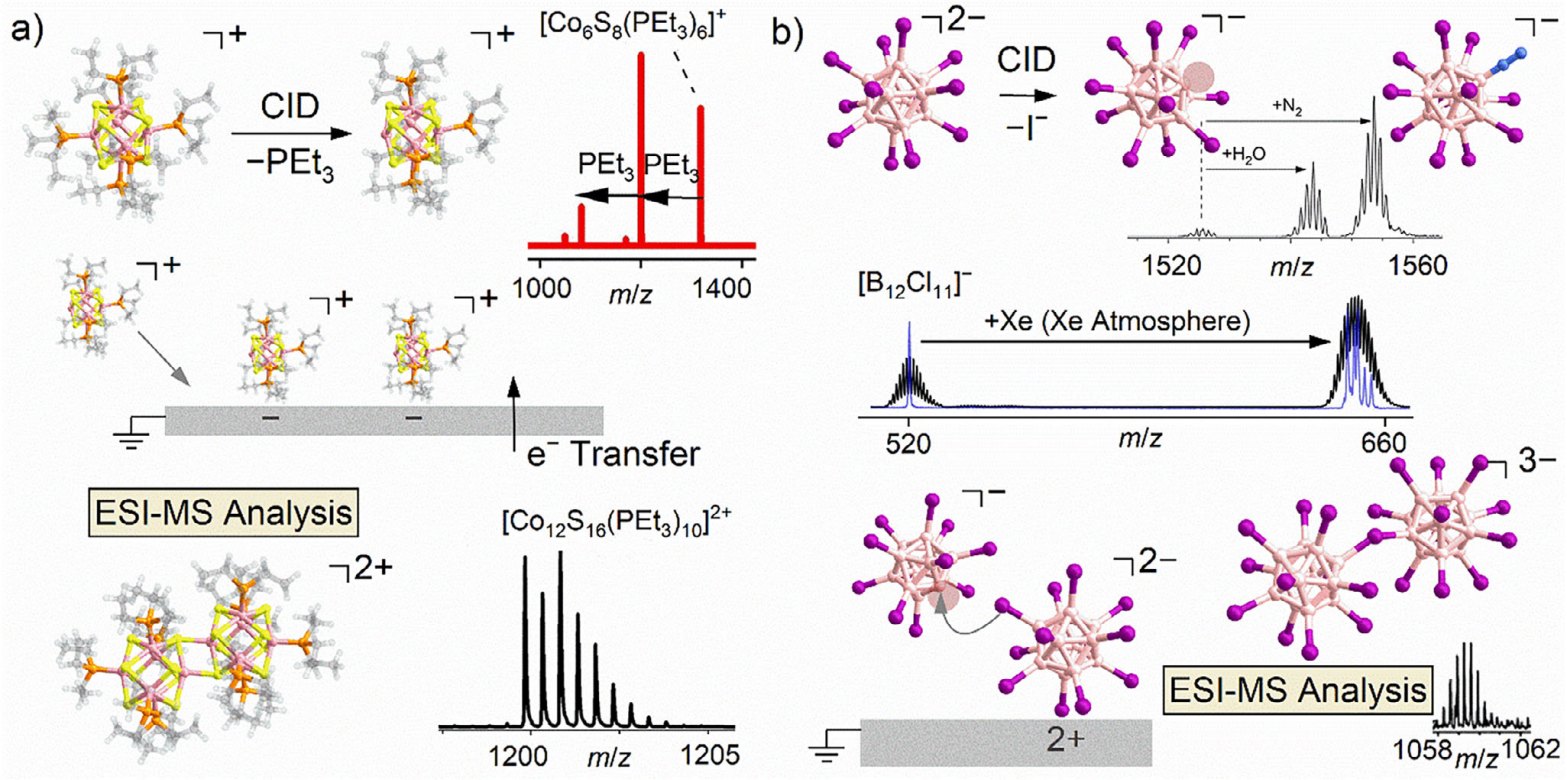
(a) Generation of the fragment ion [Co_6_S_8_(PEt_3_)_5_]^+^ by elimination of PEt_3_ upon CID from the precursor [Co_6_S_8_(PEt_3_)_6_]^+^. The fragment ion was mass‐selected and deposited on a fluorinated self‐assembled monolayer (FSAM). A highly selective formation of the dimer [Co_12_S_16_(PEt_3_)_10_]^2+^ was observed when the surface layer was subsequently dissolved for MS analysis. Reprinted (adapted) with permission from Ref. [27]. Copyright 2024 American Chemical Society. (b) Top: Generation of the highly electrophilic fragment ion [B_12_X_11_]^−^ by elimination of X^−^ upon CID from the precursor [B_12_X_12_]^2−^. In an ion trap, the exceptional reactivity of this fragment becomes apparent through the observation of abundant H_2_O and N_2_ adducts with residual gas. Data is available under the terms of the Creative Commons Attribution 3.0 Unported License CC BY and reproduced from Ref. [33] with permission from the Royal Society of Chemistry. Deliberately co‐mixing Xe gas to the background atmosphere of an ion trap leads to the detection of [B_12_X_11_Xe]^−^ (here: X = Cl), as indicated by the characteristic isotopic pattern of Xe that can be observed when only ions with a single nominal *m*/*z* value are isolated from the broad isotopic distribution of [B_12_Cl_11_]^−^ in the Xe atmosphere. Data is available under the terms of the Creative Commons Attribution Non‐Commercial License CC BY‐NC and published in Ref. [34]. Bottom: Deposition of [B_12_I_11_]^−^ onto a layer containing [B_12_I_12_]^2−^ leads to the formation of the triply charged dimer [B_12_I_11_‐B_12_I_12_]^3−^ in an anion–anion reaction facilitated by the charge‐balancing environment of the grounded deposition surface. Data is available under the terms of the Creative Commons Attribution Non‐Commercial License CC BY‐NC and published in Ref. [35]. Vacant boron sites are marked with a circle.

Furthermore, **restriction 2 (changes in the nature of ions upon deposition)** needs to be considered: reactivities of fragment ions in the gas phase can only be directly correlated with reactivities in the deposited layer if the reacting ionic species is identical in both cases. This seemingly trivial statement must be emphasized because the charge state of mass‐selected ions at the time of bond formation on the surface often remains unclear. Charge‐balancing processes, such as electron transfer from the surface, can (temporarily) neutralize cationic fragments and thus alter their reactivity. Additionally, non‐covalent interactions with the surface or other substances within the layer can change the deposited fragment ion both structurally and electronically. Such influence is often reversible and not visible in MS investigations of the layer because the layer is dissolved for ESI‐MS. Charge‐reduced ions may be reoxidized prior to detection. Although the reactions of the bpy complexes comply with both rules (A) and (B), indications for a layer thickness‐dependent charge reduction have been reported in the case of the Ni complex [32]. An interesting influence of charge‐balancing processes on reactions of deposited fragment ions is the case of [Co_6_S_8_(PEt_3_)_5_]^+^ ions (generated via CID from [Co_6_S_8_(PEt_3_)_6_]^+^). These fragments exhibited highly selective dimerization reactions on surfaces, and [Co_12_S_16_(PEt_3_)_10_]^2+^ ions were detected during ex situ MS analysis of the layer [27]. A potential coupling of two fragment ions of equal polarity cannot be probed in the gas phase, because the Coulomb repulsion separates such ions efficiently. It was assumed that the cations do not react directly with each other on the surface, but neutralization of deposited cations plays an important role in dimerization, see Figure 2a. Reoxidation, which may occur upon contact with air or solvent, would result in MS detection of the dication. Ion–molecule reactions in the gas phase cannot provide any direct probe for such a reactivity on surfaces.

Exceptionally reactive ions, which were intensively studied in both gas phase and deposition experiments, are fragments of the *closo*‐dodecaborate anions [B_12_X_12_]^2−^ (X = halogen, CN) [25, 34, 36, 37, 38]. CID yields [B_12_X_11_]^−^ with exceptionally electrophilic/Lewis acidic reactivity as demonstrated by binding to noble gases at room temperature (see Figure 2b). The most reactive ion is [B_12_(CN)_11_]^−^, which is formed from the most electronically and structurally stable precursor [B_12_(CN)_12_]^2−^ [25], in agreement with rule (A). In deposition experiments using [B_12_I_11_]^−^, triply charged dimers were found in mass spectrometric investigations, when deposited into a [B_12_I_12_]^2−^ containing layer. The very high electrophilicity/Lewis acidity probed by ion–molecule reactions (noble gases binding) rationalizes the nucleophilic attack of an iodine substituent at the vacant boron atom of the fragment ion.

**FIGURE 3:**
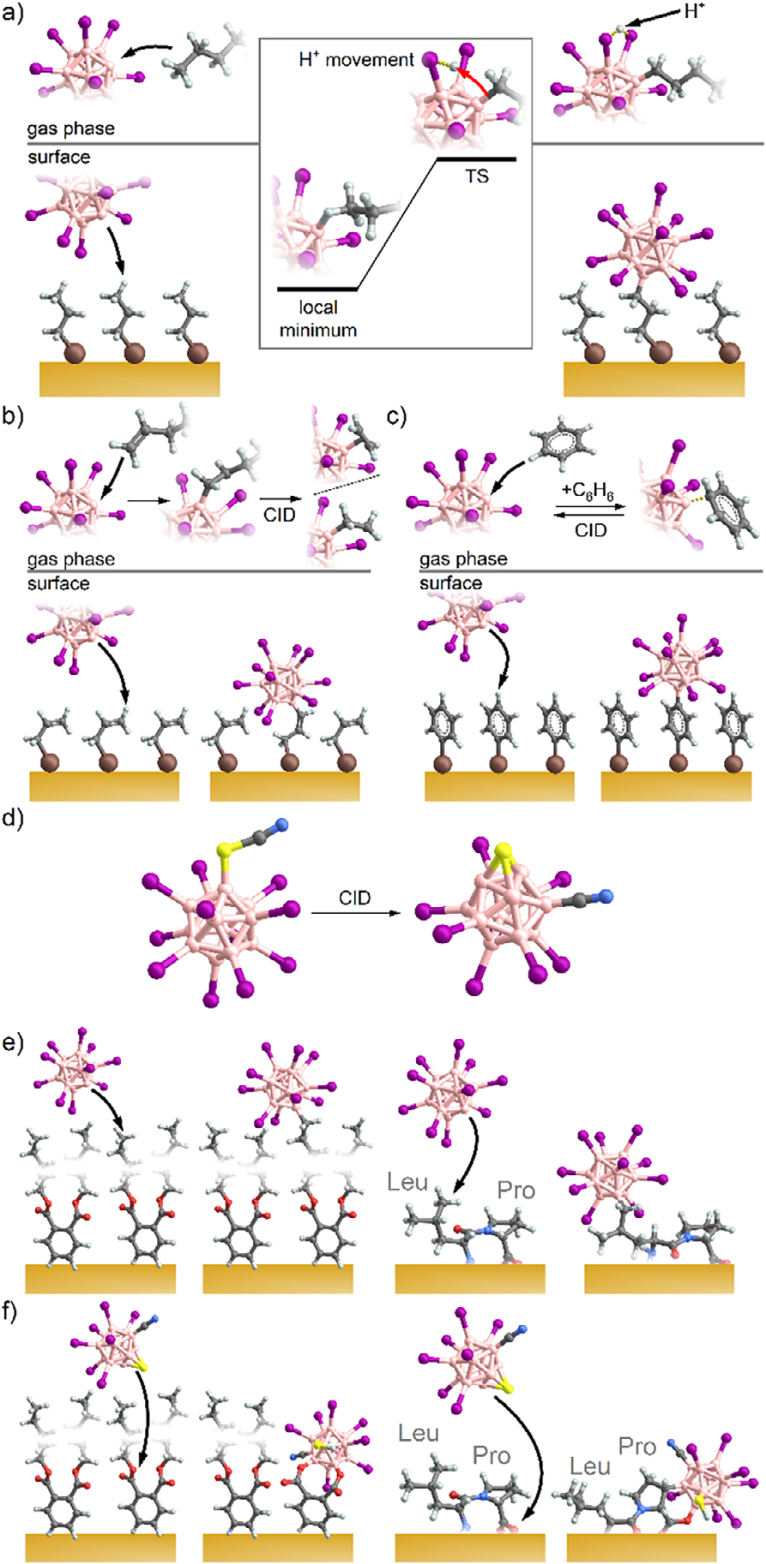
Generation and reactivity of dodecaborate fragment ions in the gas phase and at interfaces: (a) Reaction of the electrophilic anion [B_12_X_11_]^−^ with saturated hydrocarbons in the gas phase and with alkyl‐groups at interfaces. (b) [B_12_X_11_]^−^ binds to olefins via direct addition to the C═C double bond, the product yields [B_12_X_11_(C_2_H_4_)]^−^ upon fragmentation; on surfaces, H^+^ is substituted. (c) Aryl compounds add to [B_12_X_11_]^−^ in the gas phase but dissociate into the reactants upon CID; on surfaces, H^+^ is substituted. (d) Formation of the fragment ion [B_12_I_8_S(CN)]^−^ via CID from its precursor [B_12_I_11_SCN]^2−^. (e) Reaction of [B_12_X_11_]^−^ with long alkyl chains of background phthalate molecules at the layer interface (left) and with the dipeptide leucyl proline (LeuPro, right); H^+^ is substituted at the alkyl moiety. (f) Reaction of [B_12_I_8_S(CN)]^−^ in equivalent experiments as shown (e) results in functionalization at the ester groups of the phthalates (left) and predominantly the C‐terminal carboxyl groups of the dipeptide.

[B_12_X_11_]^−^ ions react with alkanes through the substitution of a proton, forming a dianion: [B_12_X_11_]^−^ + H_3_C‐R → [B_12_X_11_‐H_2_C‐R]^2−^ + H^+^. In the gas phase, the products are detected as an overall singly negatively charged ion pair. The mechanism of these ion–molecule reactions was elucidated using gas‐phase ion spectroscopy and computational chemistry [39, 40]. The mechanism appears to be directly transferable to the condensed phase for reactions with saturated alkyl chains of organic molecules, as schemed in Figure 3a. The previously discussed **restrictions 1 and 2** do not likely apply since the type of reagents is very similar (alkyl group) and the electronically stable anions are not neutralized prior to bond formation of the highly reactive vacant boron atom. The substituted proton is mobile in the condensed phase and can separate from the dianion. This results in the detection of [B_12_X_11_‐H_2_C‐R]^2−^ ions in MS analysis of the layers. Although direct transferability of gas‐phase reactions to the surface appears valid for saturated alkanes, the situation changes if the hydrocarbon is unsaturated, as schemed in Figure 3b: for alkenes, the direct binding of the [B_12_X_11_]^−^ ion to the double bond competes with the substitution of a proton. In the gas phase, binding to the double bond appears to occur almost exclusively and results in an adduct containing a Coulomb‐stabilized carbocation: [B_12_X_11_]^−^ + H_2_C═CH‐R → [B_12_X_11_^(2−)^‐H_2_C–^(+)^CH─R]^−^ (with R containing at least one hydrogen atom). CID of the adducts results (seemingly independent of the alkene structure) in the formation of [B_12_X_11_(C_2_H_4_)]^−^. For 1‐alkenes, fragmentation likely occurred via simple hydrogen rearrangement followed by the elimination of the respective alkene, whereas for other alkene isomers, the rearrangement is expected to be more complex [40]. In the case of benzene, adduct formation is observed in the gas phase, but CID results in dissociation into the reactants. In contrast, when [B_12_X_11_]^−^ reacts with alkenes or aromatic units at the interface of a surface layer, proton substitution occurs and products of the type [B_12_X_11_‐(alkene/aryl‐H)]^2−^ are detected by MS [41, 42]. The observation of proton substitution in the condensed phase despite another binding mode in the gas phase can be understood using qualitative models: The separation of the ion pair formed by proton substitution—which is impossible in the gas phase—strongly promotes this reaction pathway entropically and prevents any reverse reaction [43]. The generation of mobile H^+^ in the layer may be furthermore favorable for addressing local charge imbalance at the interface during anion deposition. Therefore, **restriction 3 (emergence of new reaction pathways in condensed phases)** needs to be imposed: Enthalpy and entropy effects of the condensed phase, which can shift competing reaction pathways in favor of an alternative reaction channel, must be considered.

The weaker electrophilic fragment ion [B_12_I_8_S(CN)]^−^ was generated by the CID from [B_12_I_11_(SCN)]^2−^ by successive loss of an iodide and two iodine atoms. In this fragment ion, a sulfur is bridging three boron atoms as depicted in Figure 3d. [B_12_I_8_S(CN)]^−^ does not react with alkanes in the gas phase but shows binding to polar groups of organic molecules like amines and sulfides. In deposition experiments, the fragment ions [B_12_I_11_]^−^ and [B_12_I_8_S(CN)]^−^ exhibit different chemoselectivities as schemed in Figure 3e, f. On the example of phthalate molecules at the interface, it was shown that [B_12_I_11_]^–^ reacts with the alkyl groups by substituting a proton, while [B_12_I_8_S(CN)]^−^ reacts with polar functional groups [33]. This reaction behavior was employed for the chemoselective functionalization of peptides on surfaces [41]. Since computational chemistry results show that the binding of polar functional groups by [B_12_I_11_]^−^ is enthalpically favored and polar molecules such as water are readily bound by [B_12_I_11_]^−^ in ion–molecule reactions in the gas phase, the very high selectivity toward alkyl group binding in deposition experiments is remarkable. Contrary to intuition, the high selectivity can be traced back to the high reactivity of this fragment, which reacts via “first contact” when it arrives at the layer interface. Orientation of the organic reaction partners at the layer‐vacuum interface is driven by the nonpolar nature of alkyl residues oriented toward the vacuum (similar to a surfactant) and is considered as **restriction 4 (orientation effects at interfaces)**. This last restriction plays an important role in the case of highly reactive ions that react even with nonpolar groups upon contact. The alternative reaction of the [B_12_I_11_]^−^ ion with polar groups in surface layers was enabled using structurally “rigid” coordination polymers that anchor polar groups at the vacuum interface [44].

## 3 Ion–Molecule Reaction Products as Building Blocks for New Compounds

**FIGURE 4:**
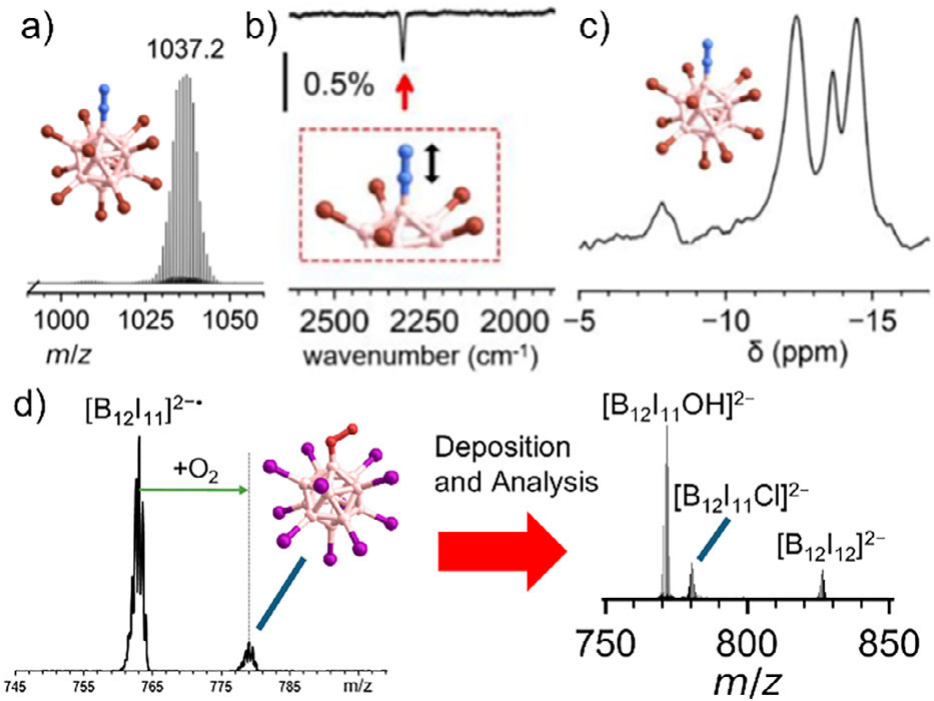
Deposition of ion–molecule reaction products can lead to the accumulation of the intact product, like in the case of [B_12_Br_11_N_2_]^−^, which was subsequently analyzed ex situ by (a) analytical MS of the dissolved surface layer, (b) reflection–absorption infrared spectroscopy of the surface showing the N─N stretching vibration, and (c) ^11^B NMR spectroscopy. Data is available under the terms of the Creative Commons Attribution Non‐Commercial License CC BY‐NC and published in Ref. [42]. In contrast, gas phase reaction products like (d) [B_12_I_11_O_2_]^2−•^ can undergo follow‐up reactions in the surface layer (containing water and hydrocarbons along with the deposited anions), yielding stable products like [B_12_I_11_OH]^2−^ as the main product, as indicated by analytical MS of the dissolved layer. The gas phase mass spectrum showing O_2_ addition to [B_12_I_11_]^2−•^ is reprinted from International Journal of Mass Spectrometry, 436, J. Warneke et al., Gas phase fragmentation of adducts between dioxygen and *closo*‐borate radical anions, 71–78. Copyright 2019, with permission from Elsevier.

The previous section emphasized that ion–molecule reactions in the gas phase can lead to different binding modes than found for the reactions of the same mass‐selected fragment ions with similar reagents at interfaces. This renders the direct application of ion–molecule reaction products interesting for mass‐selected deposition. Furthermore, ion–molecule reactions in the gas phase enable the binding of gaseous reactants to fragment ions, which do not adsorb at interfaces at room temperature. The deposition of complex ion–molecule reaction products has only sporadically been investigated so far. Figure 4 shows analytical results of a layer generated by deposition of mass‐selected [B_12_Br_11_N_2_]^−^ generated in an ion–molecule reaction from [B_12_Br_11_]^−^ and N_2_ [42]. The product was characterized using conventional analytical methods (MS, IR, and NMR spectroscopy), demonstrating the small‐scale synthesis of a new substance using a mass‐selected ion–molecule reaction product. The bound N_2_ in the stable product ion (DFT‐calculated attachment enthalpy: −150 kJ/mol [B3LYP‐D3BJ/def2‐QZVPP]) [34] can also be seen as a protective group for the reactive binding site of the fragment ion. Heat‐induced release (225°C for 2 h) generated the reactive [B_12_Br_11_]^−^ within the layer, which then attacked co‐deposited counterions [42]. Another interesting scenario is the formation of a reactive species in an ion–molecule reaction and subsequent deposition onto surfaces. The radical fragment ion [B_12_I_11_]^2−•^ binds O_2_ in the gas phase [45]. Mass selection and deposition of the product [B_12_I_11_O_2_]^2−•^ results in the formation of [B_12_I_11_OH]^2−^ on the surface, with minor impurities due to [B_12_I_11_Cl]^2−^ (contamination in the precursor salt with a similar *m*/*z* as [B_12_I_11_O_2_]^2−•^) and [B_12_I_12_]^2−^ that are likely co‐deposited, as shown in Figure 4.

## 4 Conclusion and Outlook

Ion–molecule reactions in the gas phase provide information about the intrinsic reactivity of fragment ions and can be used to probe bond formation with reagents in preparative mass spectrometry. Here, restrictions for the knowledge transfer from gas phase chemistry to the interface chemistry of reactive ions are discussed. In published examples, we have shown that

1. comparability of reaction partners,
2. changes in the nature of ions upon deposition,
3. emergence of new reaction pathways in condensed phases, and
4. orientation effects of reagents at interfaces,

have to be taken into account in order to understand that different binding modes on the surface, as compared to gas‐phase reactions with similar/identical partners, may be observed. The mass‐selected deposition of ion–molecule reaction products offers significant potential: gaseous products that cannot be obtained by conventional preparative methods can be accumulated on surfaces and used in subsequent reactions. Reactive products, including complex radical ions [46] and other reactive ions [47, 48, 49], can also be generated and probed for surface reactions.

**SCHEME 2:**
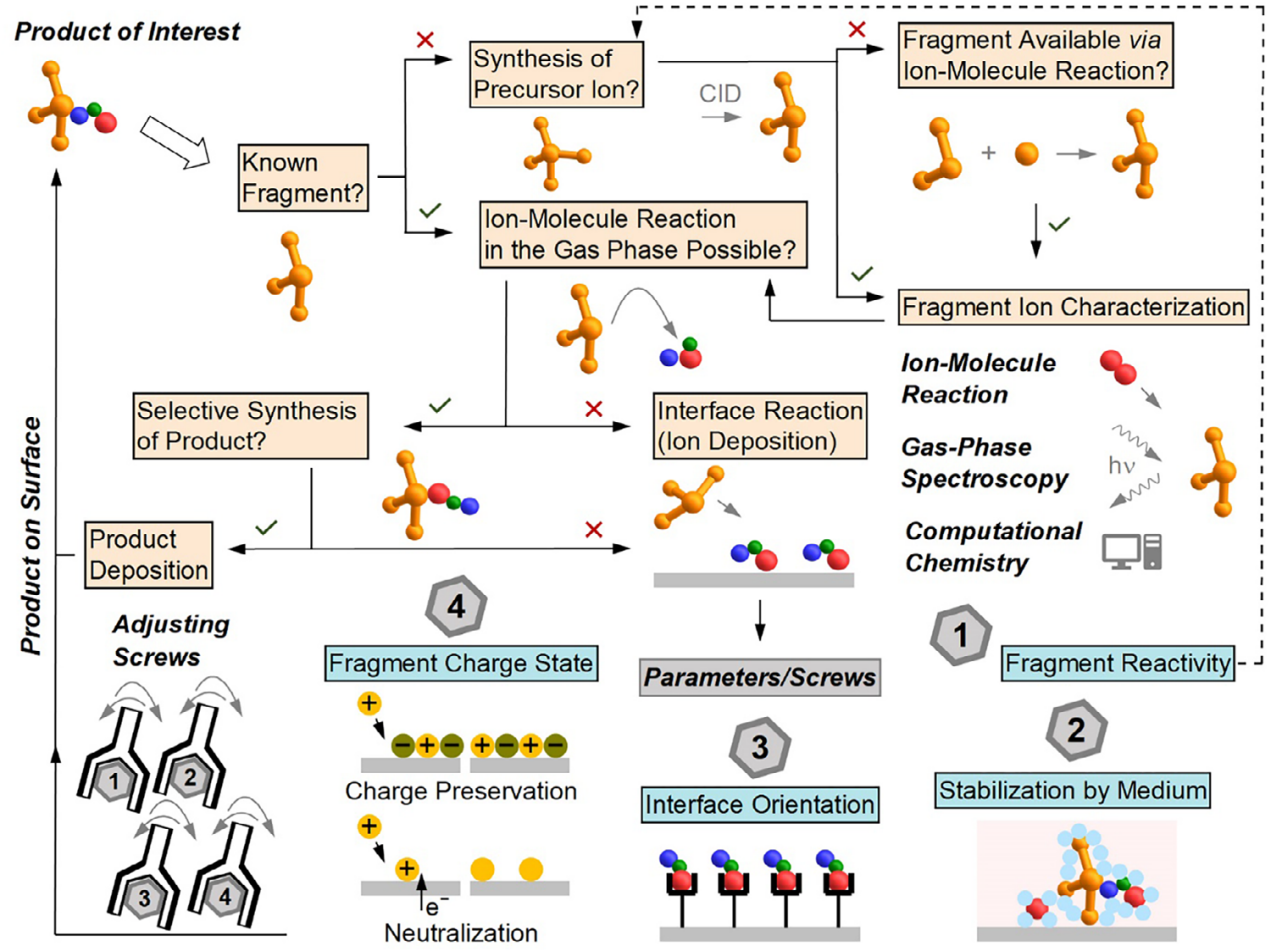
Schematic representation of a retrosynthesis‐like approach to prepare a targeted product following the conceptual foundations as introduced in this article.

Although the application of ion–molecule reaction products in preparative mass spectrometry has only been sporadically explored in recent years, there is clear potential for designing difficult‐to‐synthesize ions and molecules at interfaces. Fields including thin layer preparation in materials science [23, 24], as well as charge tagging of surface‐adsorbed biomolecules for bioanalytical applications [41], could greatly benefit from these emerging possibilities. Scheme 2 illustrates a retrosynthetic‐inspired approach for the small‐scale synthesis of new products, incorporating multiple adjustable parameters. The process begins by analyzing the product structure to identify suitable “building blocks” (fragment ions). If no suitable fragments are known from fragmentation spectra or analytical mass spectrometry literature, the synthesis of a suitable precursor may be possible. For example, a reactive carbocation may be generated from a protonated amine upon cleaving ammonia using CID. Fragmentation rules documented in mass spectrometry textbooks may provide essential guidance for this step. As an alternative, a suitable reactive “building block” can be generated through an ion–molecule reaction. For example, a reactive oxygen center can be formed by binding a radical ion to background O_2_. The design of the precursor should be adapted according to the desired reactivity and selectivity of the target fragment ion. The typical rules for the complementary reactivity of even‐electron reagents, which should selectively form a bond, apply here. For example, if a very weak Lewis base is to be bound by the fragment ion, the ion should be optimized with respect to a high positive partial charge and a lowest unoccupied molecular orbital (LUMO) localized at the binding position, for instance, by introducing electron‐withdrawing substituents. Successful enhancement of the fragment ion's Lewis acidity is usually reflected in the higher collision energies required to cleave the Lewis base leaving group (e.g., NH_3_) from the precursor. However, fragment ions can also be optimized with respect to their reaction kinetic properties. For instance, radical fragment ions intended to selectively bind a double bond in the gas phase should be “polarity‐matched” to the reaction partner [46]; for example, a nucleophilic radical ion should be used for electron‐deficient double bonds. Computational chemistry studies combined with gas‐phase spectroscopic methods provide information about the structure and electronic properties of the generated fragment ion. Meanwhile, ion–molecule reactions can directly test the reaction behavior. The rate of reactions (e.g., adduct formation with N_2_) depends on reaction parameters such as pressure and temperature in a complex way, as such parameters influence collision rates and thus the thermalization efficiencies of the “hot” collision complexes. Therefore, experiments comparing binding properties must be carried out under directly comparable reaction conditions. According to the Bell‐Evans‐Polanyi principle, an increase in the binding rate of gaseous molecules usually indicates a stronger bond formed with the neutral molecule for structurally similar fragment ions.

If a gas‐phase reaction leads to the formation of the desired product, it may be mass‐selected and deposited in layers on surfaces. If product formation in the gas phase is not possible, or if an undesired constitutional isomer is formed, the **restrictions 2–4** discussed above open new opportunities in the schemed retrosynthetic approach and may be used as “adjustable screws” for the reactions at interfaces, which are not available in the gas phase. Control over charge states, orientation at the interface, and stabilization of products may be specifically adjusted by co‐deposited counterions, surface material, thin dielectric layers, and background molecules.

## Conflicts of Interest

The authors declare no conflict of interest.
